# 3D Printing of Solvent-Free Supramolecular Polymers

**DOI:** 10.3389/fchem.2021.771974

**Published:** 2021-11-29

**Authors:** Harald Rupp, Wolfgang H. Binder

**Affiliations:** Division of Technical and Macromolecular Chemistry, Institute of Chemistry, Faculty of Natural Sciences II (Chemistry, Physics and Mathematics), Martin Luther University Halle-Wittenberg, Halle, Germany

**Keywords:** rheology, hydrogen bond, supramolecular polymer, 3D printing, polymer self-assembly

## Abstract

Additive manufacturing has significantly changed polymer science and technology by engineering complex material shapes and compositions. With the advent of dynamic properties in polymeric materials as a fundamental principle to achieve, e.g., self-healing properties, the use of supramolecular chemistry as a tool for molecular ordering has become important. By adjusting molecular nanoscopic (supramolecular) bonds in polymers, rheological properties, immanent for 3D printing, can be adjusted, resulting in shape persistence and improved printing. We here review recent progress in the 3D printing of supramolecular polymers, with a focus on fused deposition modelling (FDM) to overcome some of its limitations still being present up to date and open perspectives for their application.

## Introduction

The area of 3D printing of polymers combines different aspects in polymer science, requiring precise knowledge of molecular sciences, melt rheology, and thermal properties, as well as a detailed knowledge of inter- and intramolecular interactions. Thus, being situated within this highly interdisciplinary area, the molecular engineering of bulk properties, important for designing appropriate flow properties during the printing process, has become crucial. The area of supramolecular polymers, featuring precisely engineered intermolecular interactions, allows to reach improved material characteristics by dynamic interactions like enhanced material strength, toughness, self-assembly, and stimuli responsiveness (Moore, 1999; Brunsveld et al., 2001; Binder et al., 2007a; de Greef and Meijer, 2008; Herbst et al., 2012; Herbst et al., 2013). Both, mechanical and thermal properties of these self-assembled materials, are strongly influenced by the nature and amount of non-covalent bonds and their interactions (such as supramolecular hydrogen bonding, π-π stacking, metal complexation, and ionic interactions). (Seiffert et al., 2015). This chemistry and structural design of a supramolecular moiety influence the assembly of polymer by their strength of interaction (Herbst et al., 2010; Appel et al., 2012; Li et al., 2012; Chen and Binder, 2016; Chen et al., 2020a), in turn changing material characteristics by chemically programming the different supramolecular moieties. In recent years, it has been shown especially that the use of hydrogen bonds as supramolecular entities has allowed to tune material properties over an enormous range, especially addressing their final mechanical properties. Thus, in recent times, the field of non-covalent interactions is dominated by materials based on hydrogen bonds (Binder et al., 2007a), as the strength of these bonds can be well tuned over many orders of magnitudes, additionally allowing their comparably simple embedding into many commercial polymers known to date. Therefore the focus of this review will be placed on hydrogen-bonding polymers and their interactions in view of 3D printing and the materials generated therefrom.

Additive manufacturing of supramolecular materials relies on different techniques like material extrusion, vat polymerization, inkjet printing, and bioprinting (Melchels et al., 2010; Bajaj et al., 2014; Pedde et al., 2017). The individual printing techniques require a wide range of flow properties, and thus, supramolecular interactions offer advantages and disadvantages to engineer and adjust the printing properties in turn allowing individual design (Pekkanen et al., 2017). Traditionally, the extrusion of thermoplastics requires the melting of the polymer, making supramolecular polymers ideal candidates for direct extrusion due to their strongly temperature- and shear force-dependent supramolecular interactions. Some current limitations and problems in printing of traditional polymers, especially in fused deposition modeling and deposition-based printing, are addressed by the special functions of supramolecular polymers. Obstacles like high printing temperatures, interlayer adhesion, and sample warping can be addressed by the use of supramolecular interactions in 3D printing (Bochmann et al., 2015; Kollamaram et al., 2018; Geng et al., 2020). Besides the introduction of self-healing properties, a well-known feature of supramolecular materials is the increased material strength without relying on different filler materials, resulting in an easier processing of strong polymers in 3D printing (de Espinosa et al., 2015; Yu et al., 2017; Gao et al., 2019). As the printing of supramolecular polymer systems in solution or in gels (supramolecular hydrogels) is not considered in this review, the reader is kindly referred to the following literature for more information on hydrogel printing: Pekkanen et al., (2017); Ganguly et al. (2020a); Ganguly et al. (2020b); Chimene et al. (2020); Das et al. (2021); Kumar et al. (2021); and Madduma-Bandarage and Madihally (2021).

## 3D Printing Technologies

**FIGURE 1:**
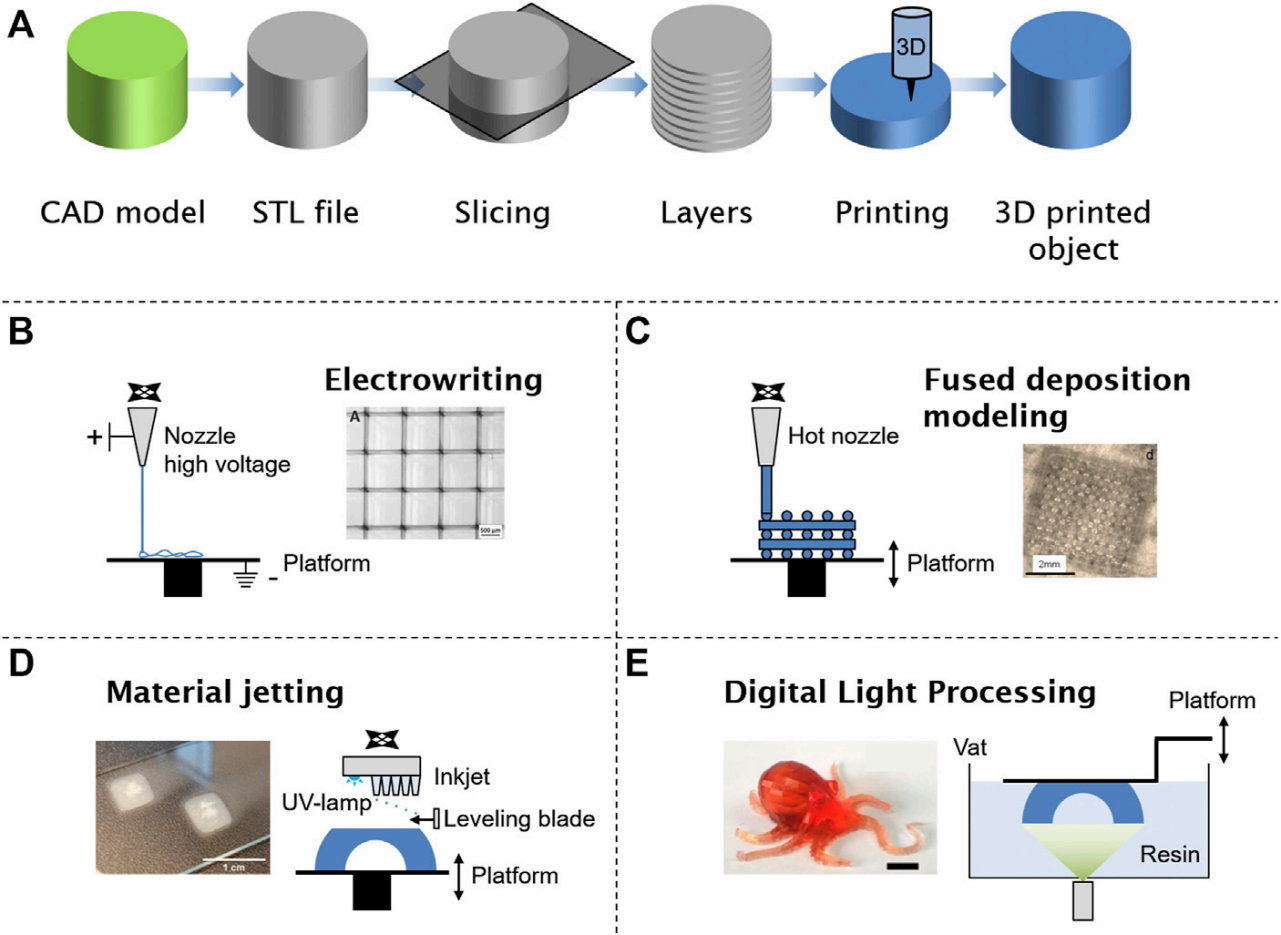
General steps for any 3D-printing technology starting with a digital model. The file is converted in an official additive manufacturing file format (STL) and sliced into several layers of some micrometers. The 3D-printing setup will print in a layer-by-layer process to obtain a 3D object **(A)**. Manufacturing of supramolecular polymers by electrospinning **(B)**, fused deposition modeling **(C)**, material jetting **(D)**, and digital light processing **(E)**. Adapted with permission (Hart et al., 2016; Hochleitner et al., 2018; Rupp et al., 2019; Zhu et al., 2021). Copyright 2018, John Wiley and Sons. Copyright 2019, John Wiley and Sons. Copyright 2016, American Chemical Society. Copyright 2021, American Chemical Society.

Three-dimensional printing is a manufacturing technique to fabricate complex structures and a large pool of different designs and geometries. Commonly, the process consists of creating a three-dimensional model by computer-aided design (CAD) software resulting in an STL file format, transferring it to the 3D printer, and manufacturing the object in a layer-by-layer approach (Figure 1A). An STL file stores information about 3D models describing the surface geometry of a three-dimensional object. Different techniques like stereolithography (SLA) (Huang et al., 2020), fused deposition modeling (FDM) (Peltola et al., 2008), powder bed fusion (Bhavar et al., 2014), material jetting (Yang et al., 2017), (melt)-electrospinning (Bachs-Herrera et al., 2021), and direct light processing (Lu et al., 2006) are well-known techniques for additive manufacturing (Figure 1B–E). In early times, 3D printers were used for designing and prototyping in small batches offering fast and cheap manufacturing, whereas in current technology, advantages of this highly developed technique are high precision, fast speed, material saving, personalized design, and production on demand. In the future, the advantages of 3D printing will be defined newly by ongoing research activities, which eliminate limitations of the technology and help understanding of fundamental processes (Ngo et al., 2018).

## Predicting Printability by Rheological Data

Rheology is an absolutely required tool to analyze new materials for any polymer fabrication technique, in particular for 3D printing, where the flow processes during printing and the thermal processes after printing are crucial. The rheological properties of manufactured materials depend on factors like the polymer glass transition; its melting temperature; the use of additives and additional composites, plasticizers, and fillers; and the dimensions of the used printing tools. The characterization of such flow properties in the melt state can then be correlated to predict hot melt extrusion. FDM uses heat to obtain a semi-molten state for a 3D-printed thermoplastic polymer, which is then pushed through a nozzle. Therefore, high temperature rheology and shearing effects are of high importance. Thus, the used polymers and filaments should have appropriate rheological properties and mechanical strength for obtaining a good processability, with viscosity being the main important parameter. As the shear rate during extrusion through a small nozzle can go up to 10^4^ s^−1^ (Cicala et al., 2018), the viscosity window of the used printing setup must be probed beforehand, after which new materials can be tested to fit inside the viscosity range to ensure their printability. Knowledge about the printing properties beforehand will thus save “trial-and-error” time and will prevent nozzle blockage or polymer dripping (Azad et al., 2020). Obstacles known for such printing processes are, e.g., when printing below the recommended temperatures, the polymer can block the nozzle (high viscosity) or lead to a low connection strength between newly formed layers after the deposition process (Yang et al., 2018). In case the viscosity of the samples is too low and they do not display elasticity, melt extrusion can result in flow/drip after exiting the nozzle (Cicala et al., 2018; Rupp et al., 2019). In the past for many commercial polymers, a well-established printing window can be determined for a specific printing setup considering the known printing temperatures (Aho et al., 2015; Yang et al., 2016; Bochmann et al., 2017).

**FIGURE 2:**
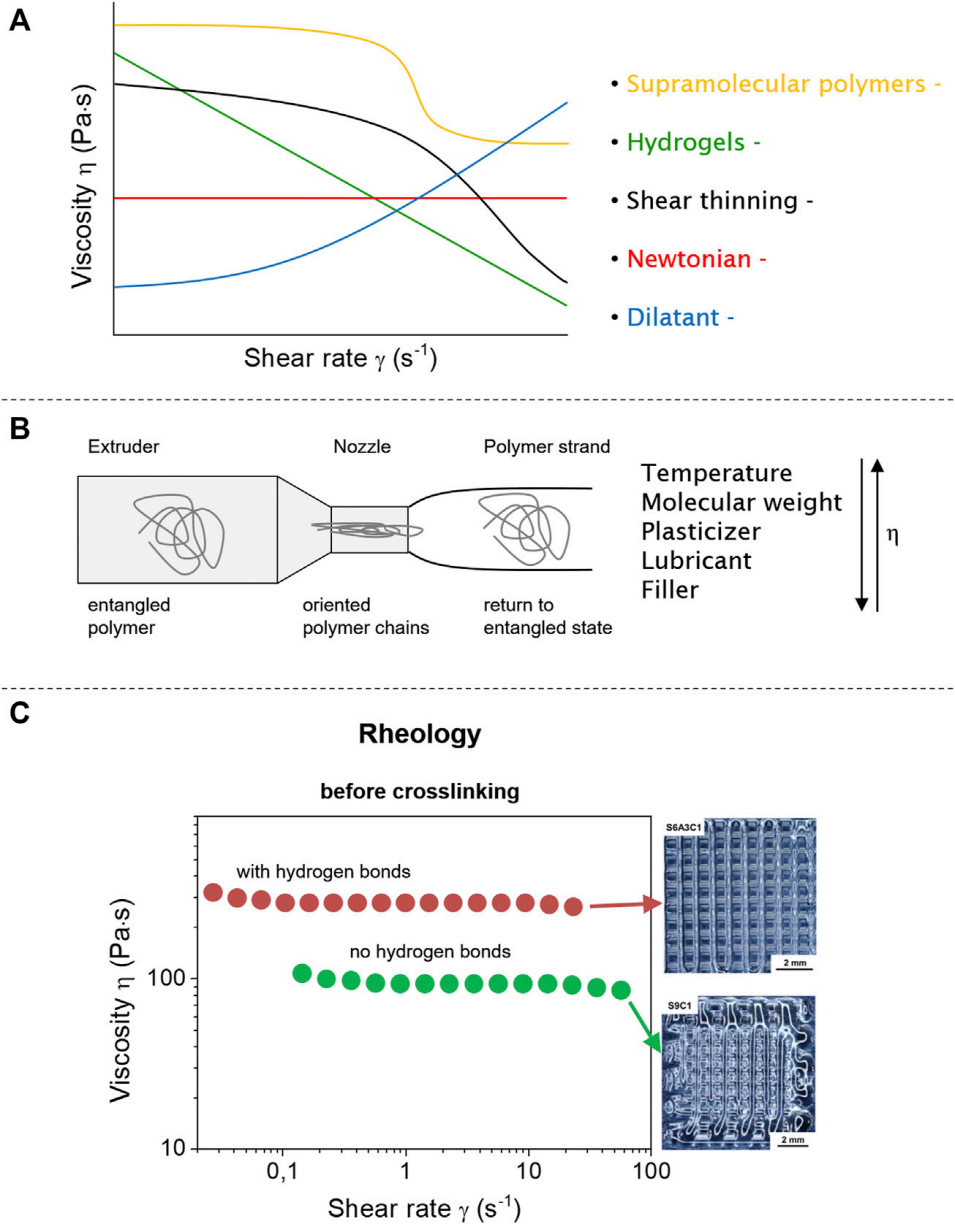
Flow behavior of different fluid systems (Newtonian, dilatant, and shear thinning) and polymer materials (supramolecular polymers and hydrogels) under shear force **(A)**. Polymers will be under shear stress during extrusion that leads to deformed and oriented polymer chains **(B)**. Supramolecular interactions change the flow properties compared to neat polymer materials improving the form stability after extrusion **(C)**. Reproduced with permission (Liu et al., 2020). Copyright 2020, American Chemical Society.

Using rheology, the melt flow and shear thinning behavior of polymers is determined, especially as long polymer chains often show shear thinning behavior when subjected to higher shear forces (Wang et al., 2013). They then display properties of viscous liquids and elastic solids depending on the deformation, the temperature, and time: a so-called viscoelastic material (Vlachopoulos and Strutt, 2003). For the rheological properties, there are two types of flow for non-Newtonian liquids: there is a simple shear flow (easy to determine) and extensional flow (pressure-driven flow) taking place in extrusion and injection molding (Aho et al., 2015), with viscosity being the most important to characterize the flow in relation to applied shear force (Figure 2A). Whereas ideal Newtonian fluids show a constant viscosity for any shear rate, polymers are non-Newtonian fluids and show shear thinning behavior based on entangled chains being disentangled and oriented along the force-field. A typical shear viscosity curve of a polymer thus displays different regions depending on the shear rate. Starting with low shear rates, a plateau with Newtonian behavior is formed where the polymer chains are still entangled. With increasing shear rate, the polymer chains get oriented and disentangled (Figure 2B), resulting in a reduction of viscosity. When most of the polymer chains are disentangled, a secondary Newtonian plateau is formed, which is often difficult to measure experimentally for polymer melts (Aho et al., 2015; Larson and Desai, 2015). For comparison, typical fluids like ideal Newtonian liquids, dilatants, supramolecular polymers (Folmer et al., 2000; Herbst et al., 2010; Seiffert and Sprakel, 2012), and hydrogels (Patel et al., 2005; Shao et al., 2015; Chen et al., 2017a) are shown as well (Figure 2).

The viscosity of the polymer melt is strongly changed by temperature (Dobrescu and Radovici, 1983; Wang and Porter, 1995), as long polymer chains have different movements (rotation, different conformations, and side groups), also inhibited by entanglements or loops present in their surroundings. All these effects and close packing of chains in the polymer melt affect the movement of single chains. However, the most important parameters for the extrusion process in FDM are on the side of the printer, mainly the temperature and the extruder screw speed (Mackay, 2018). If the viscosity of the polymer melts increases, the torque of the extruder is strongly moving towards higher values, reaching values where the printability is rendered impossible. This increase in viscosity can be reversed by higher temperatures, always in view of the polymer’s decomposition temperatures, which have to be considered separately (Aho et al., 2015; Mackay, 2018). It must be kept in mind that for shear thinning polymers an increase of rotation speed helps to decrease the viscosity (Liang, 2002), with the disadvantage that if a polymer displays too low a viscosity, it will lose its shape after extrusion, thus rendering the 3D-printing process useless.

Thus for an optimal extrusion of an unknown polymer material, the relation between the three parameters temperature, viscosity, and shear rate has to be analyzed (Figure 2C) (Ramanath et al., 2008). Subsequently, the rheological behavior is related to the polymer behavior during extrusion, where the polymer chains are subjected to a nozzle-dependent shear force inside the extruder, often experiencing an orientation along the extrusion direction (Figure 2B) (Aho et al., 2015). Furthermore, thixotropic properties also play a role in extrusion-based 3D printing, recovering its properties after an applied shear force. For a perfect printing result the polymer should regain its initial properties very fast after it has experienced the shear force directly after deposition before further layers are added (thus forming self-supporting layers) (Conceição et al., 2019; Kim et al., 2019). Thus, even as the true viscosity of polymer melts during FDM (inside a printing nozzle) is not measurable, the toolbox of rheology measurements helps to understand the behavior of the printed materials. For a given printing setup, the rheological data offers analytical information for suitable polymers and composites (Rahim et al., 2019). Measurements, next to viscosity, provide information about layer adhesion, elasticity, shear thinning, and thixotropy.

## Rheology of Supramolecular Materials

The melt rheology of supramolecular polymers is far more complex than the rheology of commercial thermoplastic polymers as additional superstructures form, which display a strong thermo-reversible and shear-dependent behavior. The modified polymers undergo multiple relaxation processes during different shear rates and temperature ranges. Supramolecular polymers with a plateau in the storage modulus G′ at low frequencies are interpreted as a rubbery plateau, where multiple associations of the supramolecular moieties take place, forming a dynamic network being based on, e.g., hydrogen bonds. When supramolecular groups are introduced into the polymer chain, their properties change on multiple levels: the association behavior of the supramolecular groups, their chain dynamics, the reversible crosslinking, and other effects such as the effective chain length or phase separation (Folmer et al., 2000; Herbst et al., 2010; Herbst et al., 2012; Reppel et al., 2013; Golkaram and Loos, 2019).

**FIGURE 3:**
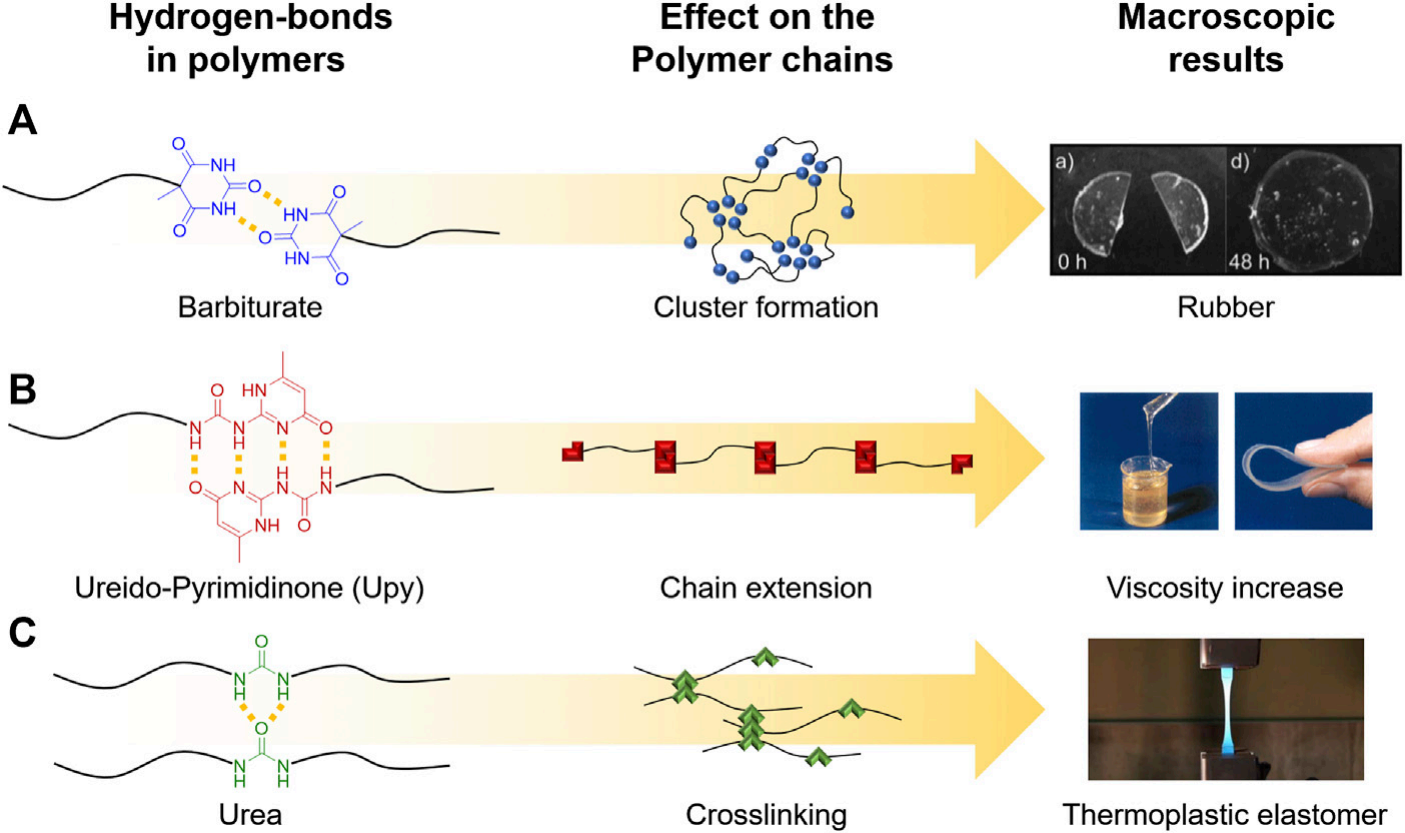
Cluster formation of the barbiturate aggregates in bivalent telechelic poly-isobutylene (PIB)–barbiturate forming a dynamic network **(A)**. Chain extension in telechelic ureidopyrimidinone (UPy)-modified polymers and their effect on viscosity **(B)**. Hydrogen bonds inside the polymer chain lead to non-covalent crosslinking to obtain thermoplastic elastomers **(C)**. Adapted with permission (Folmer et al., 2000; Herbst et al., 2012; Reppel et al., 2013). Copyright 2012, The Royal Society of Chemistry. Copyright 2000, John Wiley and Sons. Copyright 2013, Technische Mechanik.

In this context, the rheological profiles of supramolecular polymers are becoming significantly more tunable when compared to “conventional” polymers, as the supramolecular bonds can be well activated by temperature. An important example is represented by polymers containing telechelic barbiturate groups, which form hydrogen bonds in multiple directions based on the angled direction of the bonds (Figure 3A, B). These supramolecular arrangements of the H-bonds, attached covalently to the end of the polymer chains, result in a strongly thermo-reversible behavior of the hydrogen bonds, forming nano‐sized micellar clusters, which then organize into a dense supramolecular network of interconnected aggregates (Yan et al., 2014). In contrast to a covalent network, the dynamic character of the attached supramolecular bonds enables macroscopic flow of the polymer on a longer timescale depending on the temperature, as observed with frequency-dependent rheology. Bivalent telechelic poly-isobutylene (PIB)–barbiturate polymers as shown in Figure 3A thus display a rubber-like behavior at high frequencies when compared to their flow properties at low frequencies. As a rule of the thumb, the differences in timescale can be related to the lifetime of the aggregates, where at high frequencies the lifetime of an aggregate is much longer than the applied frequency, whereas for low frequencies, the molecular exchange between aggregates can be observed (Herbst et al., 2012). Due to the low molecular weight of individual PIB chains (below the entanglement molecular weight Mc for PIB), the plateau in the modulus is explained by formation of a dynamic network (Figure 3A). As a result, by combining rheology with published SAXS data, the aggregates at the chain ends are connected by bridging PIB chains (Yan et al., 2017).

A similar behavior, which leads to a significant virtual increase of the molecular weight by “chain extension,” has also been shown for other hydrogen bonds such as ureidopyrimidinone (UPy) groups, which were also shown to form additional stacked aggregates in a second dimension in telechelic poly(ethylene-co-butylene) (Kautz et al., 2006) (see Figure 3B) (Folmer et al., 2000). Thus, the chain length of the telechelic polymers is increased by the association of the UPy groups, leading to a virtually increased molecular weight, which can be reduced by applied shear forces, e.g., during FDM by temperature- or shear force-induced rupture. After printing (and relaxation), the chain-extended structures are reformed, leading to structural stability and improved adhesion between the printed layers. Moreover, it was proposed that an additional stacking of the UPy groups is supported by hydrogen bonding of the urethane/urea groups next to the UPy moieties (Van Beek et al., 2007; Bobade et al., 2014). An important additional contribution to the improved printability of such supramolecular “chain-extended” polymers is induced by avoiding phase segregation effects, as the UPy groups present in linear polymers (Kan et al., 2019; Houston et al., 2016) can induce phase separation, in turn changing the relaxation behavior, then behaving more like shorter chains rather than long segregated systems (Boothroyd et al., 2019; Golkaram et al., 2019). A third principle important in many supramolecular polymers is the formation of transient networks, formed by hydrogen bonds inside a thermoplastic polymer, which leads to supramolecular transient crosslinking between the chains (Figure 3C), in turn increasing melt viscosity and elasticity, able to facilitate 3D printing of such polymers (Reppel et al., 2013; Döhler et al., 2020).

## Hydrogen-Bonding Polymers

**FIGURE 4:**
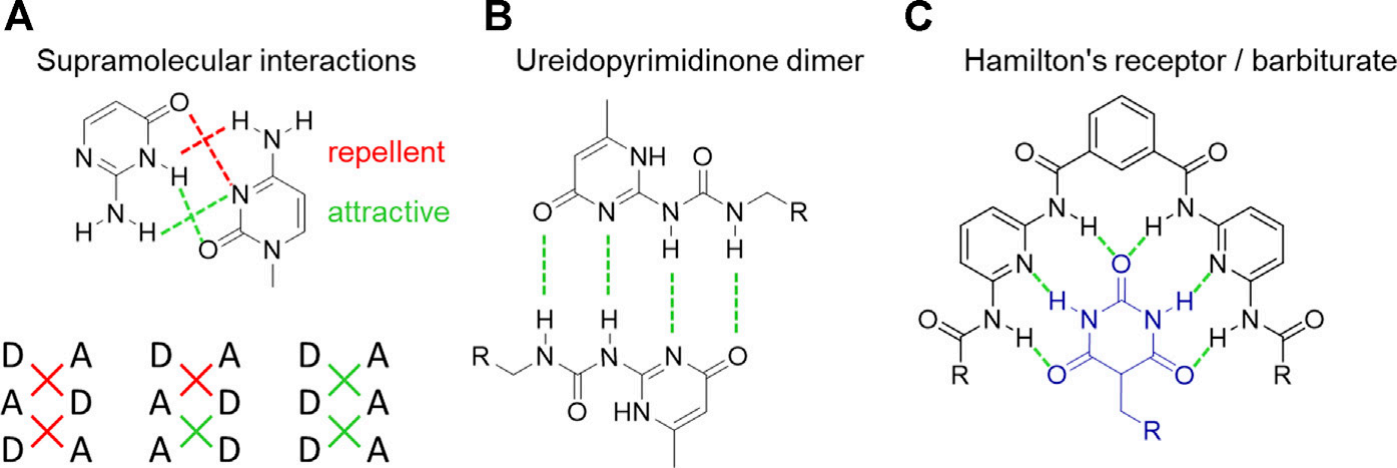
Triple hydrogen bonds bearing different donor **(D)** and acceptor **(A)** [side arrangements display different association strengths **(A)**, the ureidopyrimidinone group with an AADD bonding scheme **(B)**, and a very strong hydrogen-based interaction between Hamilton’s receptor and the barbiturate group **(C)**].

In nature, hydrogen bonds (Figure 4) play a very important role contributing to the formation of different peptide structures (α-helix and β-sheet), ligand–receptor binding, or enzymatic catalysis (Thompson and Korley, 2017; De Santis and Ryadnov, 2015). Peptide hydrogen bonds are based on the interaction between amides, urethanes, or ureas due to the presence of a proton donor (NH, general: XH or D) and a proton acceptor (C=O, general Y or A) (Brunsveld et al., 2001; Prins et al., 2001). The resulting strength of hydrogen bonding complexes is determined not only by one single interaction but also by the number of hydrogen bonds and the architecture they are arranged in. The association strength *K*_assn_ of different triple hydrogen bonds, including different donor and acceptor side arrangements (Figure 4A), is shown to depend not only on the number of hydrogen bonds but also on their spatial arrangement. The simplest case (AAA ↔ DDD) showed the highest association constant, getting weaker when exchanging one position (AAD ↔ DDA), with the last case (DAD ↔ ADA) displaying the lowest association constant (Murray and Zimmerman, 1992; Jorgensen and Pranata, 1990; Pranata et al., 1991). The effect is explained by secondary electrostatic interactions between the moieties. The interactions of diagonally linked sides show a strong attraction if they are of different kind (A ↔ D), and if they are of the same kind, they repel each other (A ↔ A, D ↔ D). These secondary attraction interactions deliver a tool for tuning supramolecular materials with only slight changes in their chemical structure. Changing the number of hydrogen bonds also allows to influence the association strength ranging from two hydrogen bonds per site (adenine/thymine) up to six hydrogen bonds (barbituric acid/Hamilton wedge, Figure 4C) (Murray and Zimmerman, 1992; Brunsveld et al., 2001; Weck, 2007; Wilson, 2007; Herbst and Binder, 2013; Yan et al., 2014).

In the melt state and when bound to polymers, hydrogen bonds induce the formation of clusters strongly different from their in-solution state (Herbst and Binder, 2013). The strong influences of hydrogen bonding groups in a polymer melt can be easily observed in PIB-based systems with thymine (Thy), barbiturate (Bar), or UPy telechelic end groups (Figure 4B) (Herbst et al., 2012; Herbst et al., 2013; Herbst et al., 2010; Yan et al., 2014; Bobade et al., 2014; Herbst and Binder, 2013; Sen et al., 2010; Döhler et al., 2015). The barbiturate hydrogen-bonded PIBs, known to form nano-sized micellar clusters, are arranged into a dense supramolecular network of interconnected aggregates mediating increased mechanical strength (Yan et al., 2017). This network formation is driven by segregation of the attached hydrogen-bonding moieties from the non-polar PIB chains, in turn connecting the polymer chains into a transient network. The achieved thermo-mechanical behavior can be tuned (Yan et al., 2017) with the terminal relaxation dominated by the opening and closing of the hydrogen bonds (see Figure 3) by forming aggregates and chain-extended or cluster-like structures. In contrast to a covalent network, the dynamic character of the attached supramolecular bonds enables macroscopic flow of the polymer on a longer timescale (Herbst et al., 2010; Rupp et al., 2019). Furthermore, the reassembly of the network structure, driven by dynamic exchanges within the nano-sized micellar clusters, additionally features multiple self-healing properties at room temperature and below (Binder et al., 2004; Binder et al., 2007b; Herbst et al., 2010; Herbst et al., 2012; Yan et al., 2017; Campanella et al., 2018).

## 3D Printing of Hydrogen-Bonded Polymers

Additive manufacturing and processing of supramolecular polymers have the advantage to support the fabricated structures and shapes. The toolbox of supramolecular oligomers and polymers can address the limitations of 3D printing such as viscosity in the melt state, anisotropic mechanic properties, adhesion between layers, or advanced functionality post-printing. Supramolecular materials were developed for their strengthening, self-healing, stress-sensing, and shape-memory properties (Colombani et al., 2005; Herbst et al., 2012; Herbst et al., 2013; Döhler et al., 2015; Jiang et al., 2017; Campanella et al., 2018; Jiang et al., 2020), and thus, the use of non-covalent bonds compared to their neat polymer counterparts improves their printing and material qualities to a high degree (Liu et al., 2020).

Thus, e.g., liquid amorphous polymers normally not being able to form self-supported structures after extrusion were easily modified with supramolecular moieties to change them into elastic rubber-like materials being mechanically stronger (Yan et al., 2014; Rupp et al., 2019). The functionality is equally distributed throughout the complete material and is introduced as a part of the polymer chain, making handling and printing much easier. Commonly in 3D printing, the increase of mechanical properties is done by manufacturing polymer–filler composites or with highly complex printing techniques (Tekinalp et al., 2014; Weng et al., 2016; Rupp and Binder, 2020; Rupp and Binder, 2021). In the following parts, the introduction of supramolecular interaction into the 3D printing field will be explored in detail, most of them being basic scientific research. Applications for supramolecular printed parts are still at a very early stage for real-life usage outside tissue engineering, but are increasing as availability of the correspondingly modified technical polymers becomes available (Pekkanen et al., 2017).

### (Melt)-Electrospinning

In the field of tissue scaffold engineering, supramolecular polymers offer the possibility for self-healing materials recovering from applied stress (Thompson and Korley, 2017), being processed by electrospinning to build up fiber constructs. Fibers are spun in a diameter range of nanometers to micrometers and allow 3D printing with ultrathin fibers (Ewaldz and Brettmann, 2019; Lannutti et al., 2007). The mechanism of electrospinning is based on inks extruded in high-voltage electric field, and it can be regarded as a “primordial” form of 3D-printing, just on a smaller scale. In electrospinning, small droplets are accelerated and stretched to form a fiber structure, if the electrostatic field force is larger than the surface tension. Being one of the early birds, electrospinning of supramolecular polymers highly depends on their interaction strength, such as the strength of supramolecular interactions to vary the viscosity to obtain droplets or fibers (Hermida-Merino et al., 2012). This approach has been utilized with a telechelic PCL-UPy_2_ polymer, compared to other techniques like solvent casting of films, compression molding, and melt spinning. Small grids were printed with strand thickness down to around 220 µm of diameter, where, due to the dynamic properties of the supramolecular polymer, manufacturing was possible below 80°C (Dankers et al., 2006; Dankers et al., 2005). With a similar technique to electrospinning, the so-called melt electrowriting (MEW), a thermoplastic elastomer, poly(urea–siloxane), was manufactured into very small structures down to 15-µm strand thickness (Figure 1B) (Hochleitner et al., 2018). The new thermoplastic elastomer is competitive in additive manufacturing and sometimes can surpass the print properties of conventional PCL, the current gold standard in high-voltage printing (Kade and Dalton, 2021). A similar approach was performed with inkjet-based 3D printing, where a supramolecular polymer system, ejected through small needles with a high shear force, forms a self-supporting structure after deposition (Figure 1D) (Hart et al., 2016).

### Fused Deposition Modeling

**FIGURE 5:**
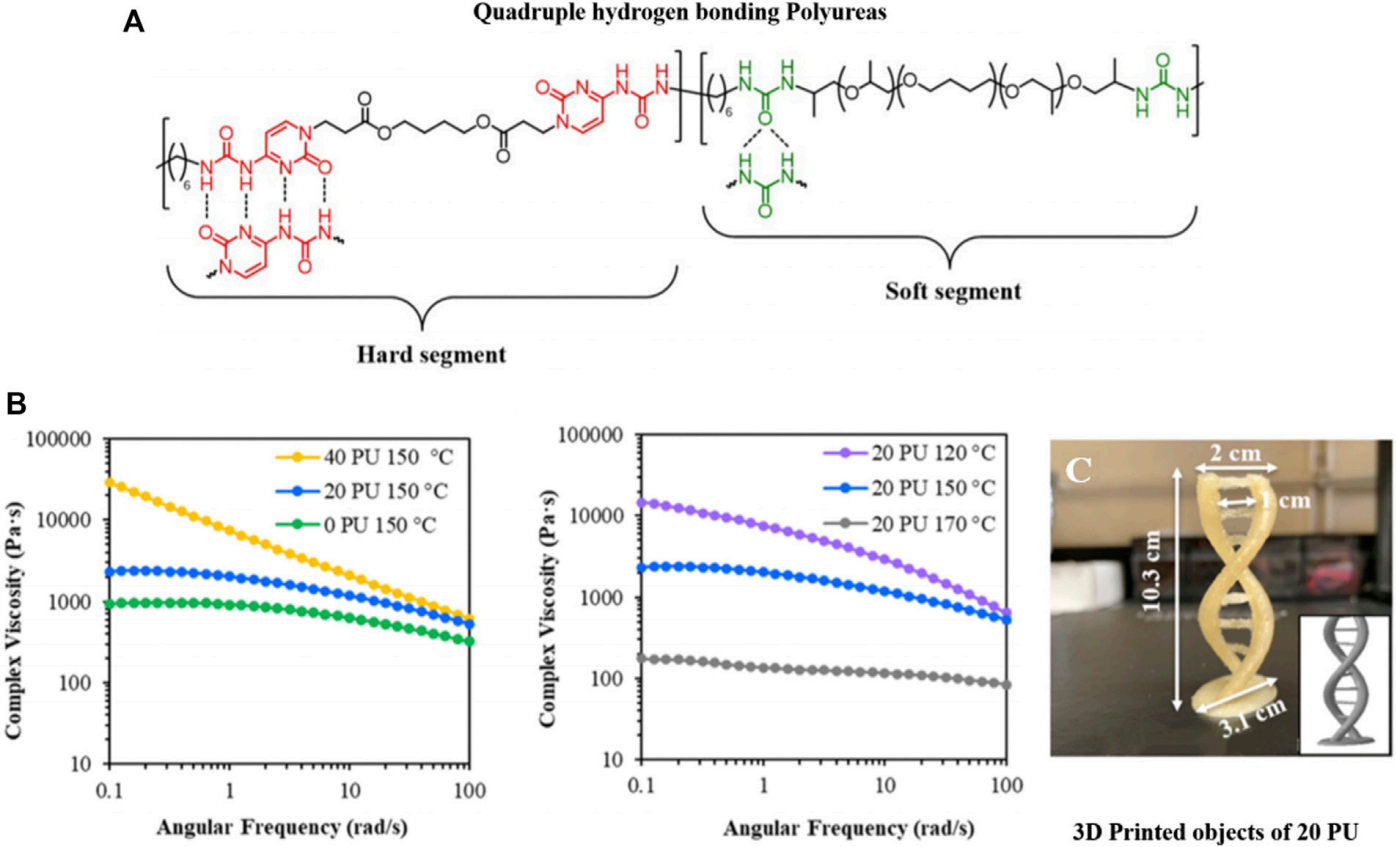
FDM-printed polyureas with UPy groups inside the polymer backbone **(A)**. The supramolecular interaction increased viscosity due to hydrogen bonding-based crosslinking and phase separation of the functional groups **(B)**. The UPy-strengthened polyurea was used in FDM printing to design a double helix **(C)**. Reproduced with permission (Chen et al., 2020b). Copyright 2020, American Chemical Society.

Chen et al. (2020b) printed polyureas with UPy groups inside the polymer backbone (Figure 5) using FDM-based methods. The supramolecular interaction increased mechanics due to hydrogen bonding-based crosslinking and phase separation of the functional groups, leading to improved adhesion between printed layers of different angles (0° and 90°) (Chen et al., 2020b). Polymers with supramolecular interactions drastically improve their mechanical properties compared to non-modified analogs (Burattini et al., 2011). The fact was proven by Liu et al. (2020) with hydrogen bonding polyesters (Figure 2C), where the materials display low printing quality due to their soft material properties, leading to spreading and flowing after the extrusion. The introduced hydrogen bonds now act as non-covalent crosslinker, improving the rheological properties for the material during printing (Liu et al., 2020). Already small amounts of supramolecular polymers or end groups change the Young’s modulus and tensile strength for polymethyl methacrylate (PMMA) polymers using FDM printing. Different ratios of PMMA to PMMA-co-UPyMA influenced the rheological and resulting printing properties and parameters (Street et al., 2019).

Rupp et al. (2019) probed the influence of the polymer chain polarity and nanoparticles on telechelic supramolecular polymers in view of FDM printing. Linear PEG and PIB polymers, equipped with H-bonds (barbiturates), were checked by melt rheology for their printing viscosity, taking into account the conditions at the nozzle and the storage tanks of a 3D printer. The PIB polymer is known to form nano-sized micellar clusters, arranged into a dense supramolecular network of interconnected aggregates (Yan et al., 2017). Printability was based on reversible thermal- and shear-induced dissociation of a supramolecular polymer network, which generates stable and self-supporting structures after printing (Figure 1C). Whereas the polar PEG–barbiturate oligomers did not form stable structures after printing, the non-polar PIB–barbiturate formed more stable structures with increased crosslinking content, underscoring the impact of phase-segregated structures to tune printability, also applicable for blends and composites, allowing to address the printability window and the form stability (Rupp et al., 2019). As expected, the temperature dependence of the relaxation time of neat PIB is considerably weaker than that of the telechelic PIB–barbiturate. The temperature dependence of the terminal relaxation time is controlled by the functional groups, rather by chain dynamics (Yan et al., 2017).

In a similar context, Döhler et al. (2020) used polydimethylsiloxane (PDMS)-based polyurea elastomers with supramolecular interactions between the polymer chains in FDM printing. Different types of polymers, especially these with hydrogen bonds in the PDMS-segments, were able to be melted at higher temperatures (up to 150°C) depending on the amount and strength of the hydrogen bonds. The printed shapes were self-supporting up to several weeks at room temperature, demonstrating the impact of the strength of supramolecular interactions on the final materials (Döhler et al., 2020).

**FIGURE 6:**
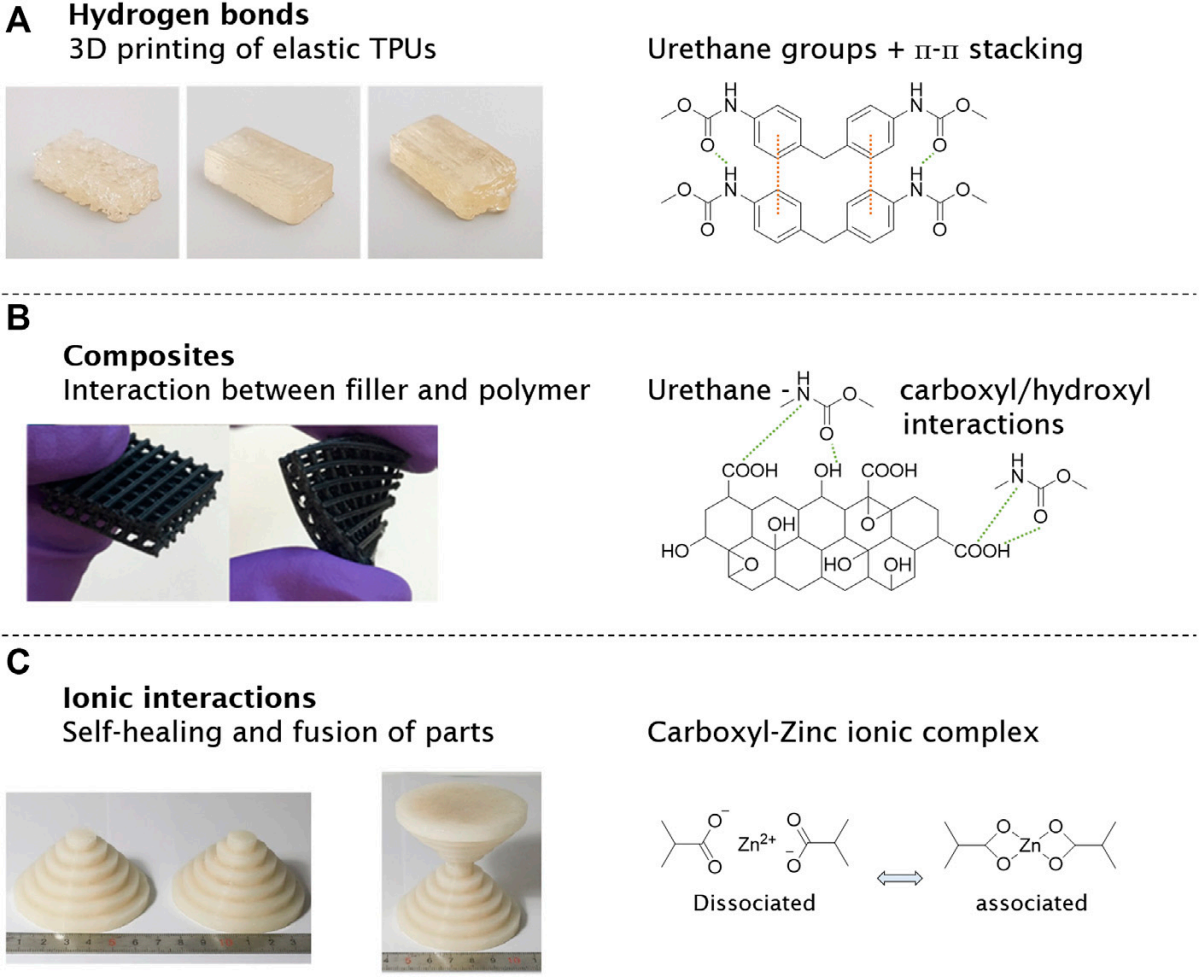
3D-printed supramolecular polymers and materials show new functionalities after manufacturing, reducing some limitations of extrusion printing. FDM printing of thermoplastic polyurethane (TPU) based on hydrogen bonds (urethane groups) and π-π stacking **(A)**. Composite materials with supramolecular interactions between polymer and graphene oxide were printed to elastic grids for tissue engineering **(B)**. An ionic-based supramolecular material was designed for its temperature-driven self-healing properties by carboxyl–zinc complexes **(C)**. Polymers reproduced with permission (Chen et al., 2017b; Lai et al., 2018; Ritzen et al., 2021). Copyright 2021, MDPI. Copyright 2017, American Chemical Society. Copyright 2018, Springer Nature.

Other polyurethane-based materials were recently manufactured by FDM featuring hydrogen bonds inside the polymer chains (Salimi et al., 2020). 3D printing of elastomers *via* polyurea vitrimers was performed by Niu et al. (2021) with a heat-driven malleability using FDM. A large improvement for interlayer adhesion was analyzed by post-annealing samples at the topology–freezing transition temperature, where associative dynamic covalent bond exchange occurs. The printed polyureas are easily recycled for up to five generations (Niu et al., 2021). Thus, a self-healing thermoplastic polyurethane (SH-TPU) was transformed into a continuous filament for FDM, where the printed SH-TPU featured the absence of the visibility of individual layers in all directions when compared to a commercial printing polymers. The results demonstrate the potential of supramolecular polymers to obtain a high printing quality with advantageous interfilament connection at the optimal printing temperature (Figure 6A) (Ritzen et al., 2021). In a comparable approach, Huang et al. (2021) synthesized telechelic supramolecular PDMS oligomers with different hydrogen-bonding moieties. The UCy-PDMS3K-UCy showed a sharp transition from solid to liquid and was printed as a filament into helix tubes. Longer PDMS chains (UCy-PDMS27K-UCy) reduced the melting point to around 74°C (Huang et al., 2021).

### Light-Based 3D Printing

Digital light processing (DLP) can polymerize monomers with supramolecular groups into a large variation of materials, from soft elastomers to rigid plastics, where the polymer is crosslinked by supramolecular and ionic bonds. Within an appropriately designed copolymer, the poly(urethane methacrylate) part acts as a soft polymer featuring hydrogen bonds, with the poly(acrylic acid) part representing the rigid phase. In addition to the supramolecular interactions, ionic interactions of zinc dimethacrylate were used as crosslinker during light curing of this polymer (Kuang et al., 2018; Zhu et al., 2021). Invernizzi et al. (2018) developed a self-healing and shape-memory material based on PCL and UPy being co-crosslinked *via* methacrylate units. A mixture of both monomers and a photo initiator was used in a DLP printer, resulting in a polymer with self-repairing abilities, provided by the UPy moieties forming (thermo-) reversible supramolecular structures (Invernizzi et al., 2018; Invernizzi et al., 2019).

### Composites

Hydrogen bonds in these polymers can be used to improve the interactions in composites. Xiang et al. (2019) used FDM to fabricate sensors made of thermoplastic polyurethane and carbonanotubes. The carbon nanotubes (CNTs) were modified with carboxyl groups forming strong hydrogen bonds towards the urethane groups. In addition to the hydrogen bonds, п-п stacking between CNT and thermoplastic polyurethane (TPU) was formed, supporting the interaction and dispersion of the nanofillers and the polymer. Compared to neat TPU, the printed composite material TPU/CNT boosted the electrical conductivity and mechanical strength. Other carbon-based materials like graphene oxide (GO) were combined with TPU and polylactid acid (PLA) blends. Functional groups on the surface of the GO (carboxyl, epoxy, and hydroxyl groups) can form hydrogen bonds towards the urethane groups and carboxyl groups, resulting in a stronger connection between filler and polymer (Figure 6B). The TPU/PLA blends increased mechanical strength and thermal stability with added GO nanofillers. On top, the 3D-printed composites exhibit good biocompatibility, which is promising for tissue engineering (Chen et al., 2017b).

### Ionic Interactions and π-π Stacking

Addressing the challenge of the often poor layer connection in FDM-processed objects, Zuo et al. (2021) worked on complex polyurethane polymers with Cu^2+^ and hydrogen bonds. The Cu-DOU-CPU materials include three dynamic bonds with reversible DOU, Cu(II)-DUO, and hydrogen bonds. Depending on the synthesized polymer ratio, the materials vary from amorphous sticky properties to solid materials melting at higher temperatures around 130°C. Other supramolecular interactions based on ionic interactions or metal complexation were also used in FDM printing and further printing technologies. PDMS polymers with carboxyl groups in the backbone were crosslinked with zinc ions to form a rigid polymer composite, representing a viscous liquid at 120°C. The liquid melt was printed by FDM into various shapes, quickly solidifying upon cooling. The so-generated PDMS-COO-Zn material is highly tunable by variation of temperature, content of ionic groups, and metal/ligand ratios (Figure 6C). Increasing the temperature shifted the ionic equilibrium towards the dissociated state, making the polymer softer and more fluid. As a result, the rigid PDMS-COO-Zn polymer became reversibly malleable, healable, and processable (Lai et al., 2018). Wilts et al. (2019) reported on stereolithography of ionic monomers based on acrylates, acrylamides, and vinyls to manufacture water-soluble parts. The new polymer offers the possibility for a high-resolution support material.

Non-covalent interactions based on π-π stacking occur between aromatic rings having similar effects like hydrogen bonds. Fused deposition modeling on polymers containing aromatic groups for self-healing or shape-memory properties are rarely reported (Chen et al., 2019). Chen et al. (2019) printed a PET copolyester with phenylacetylene–phenylimide groups as side chains featuring self-healing and flame retardancy. The π-π stacking of the phenylacetylene groups crosslinks the polyester, in turn enhancing mechanical strength and favouring shape memory. The stacking effect of the aromatic rings can be used as part of the polymer chain created by polycondensation. Printing properties of the new polyester were similar to FDM of PLA, a well-known 3D-printing biopolymer (Ji et al., 2019).

## Conclusion

With the detailed understanding of reversible molecular bonds between polymers as an additional method their printability can be better tuned and adapted to the needs of a specific 3D-printing system. Thus such supramolecular bonds are well tunable in strength and dynamics and display a direct connection to their melt rheology, inherently important for FDM processes. When supramolecular entities are attached to commercial polymers, preferably thermoplastics, rheology during and after the printing process can be tuned excellently due to their thermo- and stress-induced reversibility. The far more complex morphology of supramolecular polymers, adjustable not only by choice of the bond but also by considering additional phase segregation effects and microstructure formation, can directly affect 3D printing to generate form-stable materials after printing, still displaying sufficient flow to enable the 3D-printing process. Materials strengthened by these supramolecular interactions, provide higher extrusion quality, better interlayer connection, and functionality after being printed, in addition to properties such as self-healing and vitrimeric processability. Hydrogen bonds, where a focus of this review was placed on, are particularly useful, as their strength, as well as their dynamics, can be easily tuned and introduced by polymerization chemistry. Additional material properties, such as self-healing, can be introduced by sequential FDM processes of such polymers, opening a wide range of technological applications in the future, next to current tissue engineering. Thus the 3D printing of only minuscule quantities of material on specific locations of a larger technical part will allow to introduce, e.g., the required properties such as self-healing, self-restoring properties at the location where this is truly needed. In automotive manufacturing of intensely used parts, processes can be facilitated by placing the cost-efficient materials in thin layers only on specific locations, a fact that has been proven by printing PU-based and PDSM-based polymers. The same will hold true for other technical parts, where higher stress levels are required, but only on smaller locations, also saving costs due to the 3D-printing strategies.
